# Low dose tacrolimus exposure and early steroid withdrawal with strict body weight control can improve post kidney transplant glucose tolerance in Japanese patients

**DOI:** 10.1371/journal.pone.0287059

**Published:** 2023-10-11

**Authors:** Akihiko Mitsuke, Takahiko Ohbo, Junya Arima, Yoichi Osako, Takashi Sakaguchi, Ryosuke Matsushita, Hirofumi Yoshino, Shuichi Tatarano, Yasutoshi Yamada, Hajime Sasaki, Tatsu Tanabe, Nobuyuki Fukuzawa, Hiroshi Tanaka, Yoshihiko Nishio, Enokida Hideki, Hiroshi Harada

**Affiliations:** 1 Department of Urology, Graduate of School of Medical and Dental Sciences, Kagoshima University, Kagoshima, Japan; 2 Department of Kidney Transplant Surgery, Sapporo City General Hospital, Hokkaido, Japan; 3 Department of Diabetes and Endocrine Medicine, Graduate of School of Medical and Dental Sciences, Kagoshima University, Kagoshima, Japan; University of Cambridge, UNITED KINGDOM

## Abstract

The development of diabetes mellitus (DM) after living donor kidney transplantation (KT) is a risk factor for worsening transplant kidney function, cardiac disease, and cerebrovascular disease, which may affect prognosis after KT. At our institution, all patients’ glucose tolerance is evaluated perioperatively by oral glucose tolerance tests (OGTTs) at pre-KT, and 3, 6, and 12 month (mo.) after KT. We analyzed the insulinogenic index (ISI) and homeostasis model assessment beta cell (HOMA-β) based on the immunoreactive insulin (IRI) levels to determine how glucose tolerance changed after KT in 214 patients who had not been diagnosed with DM before KT. In addition, we analyzed the body mass index (BMI) which may also influence glucose tolerance after KT. The concentration of tacrolimus (TAC) in blood was also measured as the area under the curve (AUC) to examine its effects at each sampling point. The preoperative-OGTTs showed that DM was newly diagnosed in 22 of 214 patients (10.3%) who had not been given a diagnosis of DM by the pre-KT fasting blood sugar (FBS) tests. The glucose tolerance was improved in 15 of 22 DM patients at 12 mo. after KT. ISI and IRI deteriorated only at 3 mo. after KT but improved over time. There was a trend of an inverse correlation between HOMA-β and TAC-AUC. We also found inverse correlations between IRI and an increase in BMI from 3 to 12 mo. after KT. Early corticosteroid withdrawal or the steroid minimization protocol with tacrolimus to maintain a low level of diabetogenic tacrolimus and BMI decrease after KT used by our hospital individualizes lifestyle interventions for each patient might contribute to an improvement in post-KT glucose tolerance.

## Introduction

Kidney transplantation (KT) results in an extended and better quality-of-life for patients with end-stage kidney disease (ESKD). Modern immunosuppressants consist of calcineurin inhibitors (CNI) such as cyclosporine and tacrolimus (TAC), mycophenolate mofetil (MMF), and anti-lymphocyte antibodies along with corticosteroids, which have increasingly improved KT outcomes. However, a certain rate of post KT immunological or medical complications may occur that affect graft survival and patient longevity. Post-transplant diabetes mellitus (PTDM) is one of these important medical complications [1, 2]. Kidney recipients tend to have impaired glucose tolerance caused by several factors such as post-operative obesity and chronic exposure to several diabetogenic immunosuppressants such as CNI, corticosteroids, or mTOR inhibitors. Late PTDM is thought to be due mainly to post-operative obesity, whereas early PTDM is due to administration of immunosuppressants. The reported rate of PTDM varies in many published studies and ranges between approximately 4–25% [3]. This difference in incidence is presumably related to the racial background, definition of PTDM, and the incorrect examination of pre-KT glucose tolerability. In numerous previous reports, PTDM was underestimated because PTDM was diagnosed based on whether or not there was a need for insulin or oral diabetic medication after KT. Later, diabetes was also diagnosed based on fasting blood glucose alone or hemoglobin (Hb)A1c [2, 4]. In addition, there are a few reports of glucose tolerance being assessed prior to KT, with many reports not clearly investigating whether new-onset DM occurred after KT.

In order to comply with the American Diabetes Association/World Health Organization guidelines [5], a post-operative oral glucose tolerance test (OGTT) is indispensable. An OGTT also provides additional information regarding diabetic endocrinological function, such as the insulinogenic index (ISI) and homeostasis model assessment beta cell (HOMA-β), that helps us to understand the profound mechanism of glucose intolerance and the range of blood glucose levels. The current study surveyed the real incidence of PTDM using a 75g-OGTT in a single race population in a single institution during the 12 mos. follow-up period following KT. During this period the patients received early corticosteroid withdrawal or extreme minimization protocols to lessen the side-effects of corticosteroid. The study also evaluated the clinical parameters which might be associated with an improvement in glucose tolerance.

## Materials and methods

### Study design and population

**Fig 1:**
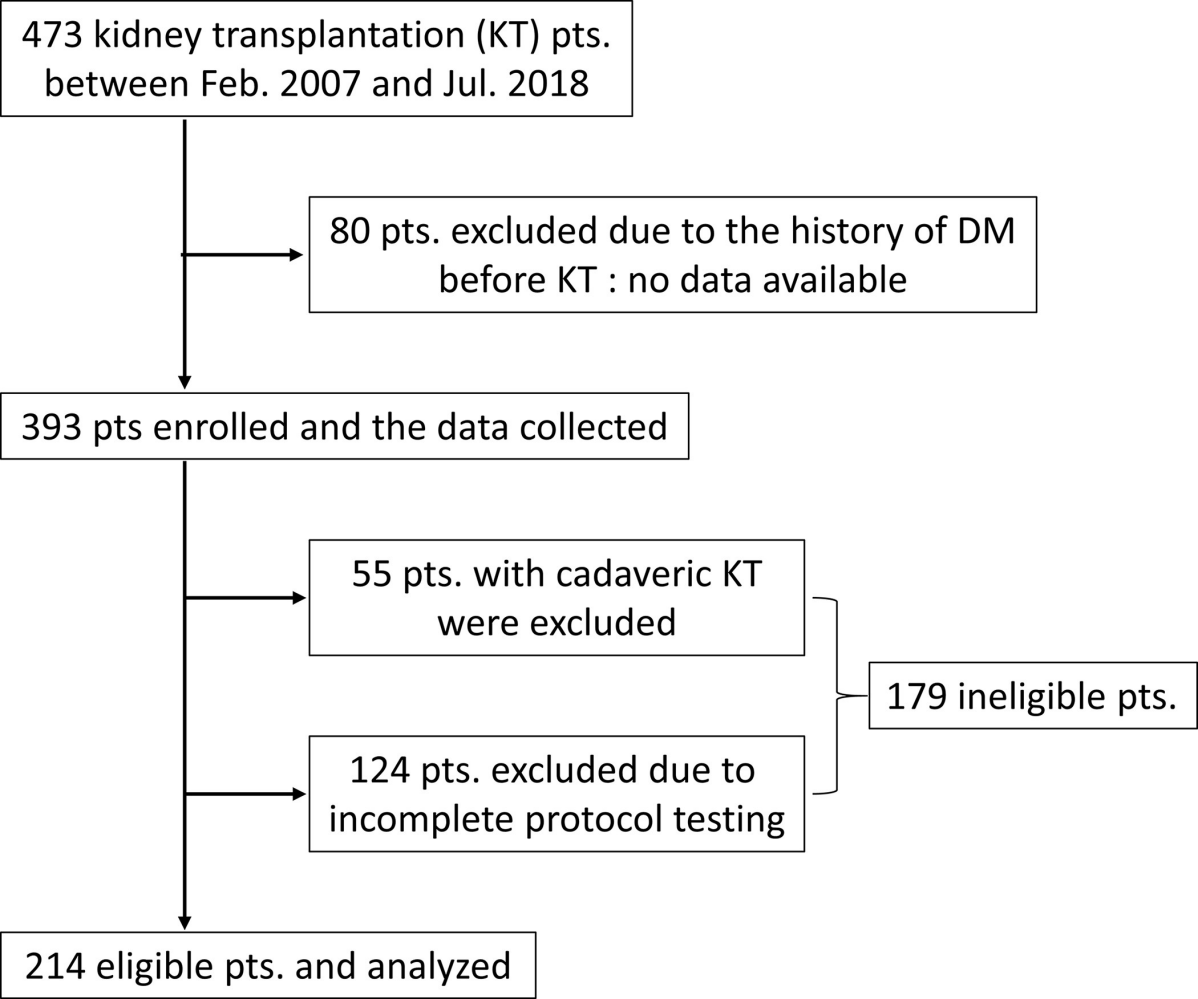
Flow diagram of the study. Eighty patients were excluded due to a treatment history of DM before KT. A further 118 patients were subsequently excluded due to incomplete protocol testing, leaving 195 patients in the final analysis.

The prospective, observational study was performed in a single center on a Japanese single race patient cohort to investigate the incidence of PTDM under early steroid withdrawal or a steroid minimization protocol with TAC. There were 473 KT patients between February 2006 and June 2018. Among them, 80 patients were excluded from the beginning due to the history of DM before KT in order to determine the actual incidence of PTDM and no data were available for them (Fig 1). Our previous pilot study demonstrated that a successful KT improved glucose tolerance in some populations (H. Harada. American Transplant Congress 2010, SanDiego: Abstract # 698). In order to thoroughly evaluate this paradoxical phenomenon the study included patients who had been diagnosed with DM for the first time by a pre-KT OGTT. We carried out 75 g-OGTTs to investigate the patients’ glucose tolerance at pre-KT and 3, 6, and 12 mo. after KT along with a protocol kidney graft biopsy and therapeutic drug monitoring using multipoint blood sampling for CNI dose adjustment. The patients (n = 55) who had cadaveric KT were excluded because OGTT tests at pre- KT or 12 mo. were skipped in the majority of them. Another patients (n = 124) who did not complete the protocol testing during the study period were also excluded from the study. Of the 393 KT patients enrolled in the study, 214 patients were included in the final analysis (Fig 1). The study protocol and informed consent documents were reviewed and approved by the institutional review boards of Sapporo City General Hospital (R04-063-954). Written informed consent was obtained from 393 KT patients. Our cohort included two minors (age of 14 and 17), and the written informed consent was obtained from their parents.

A general nutritional guidance was provided to all patients by a dietitian at pre-KT. After KT, the patients were weighed and measured at each visit. In cases with poor BMI control, nutrition guidance was again provided.

### Summary of immunosuppression protocols

**Fig 2:**
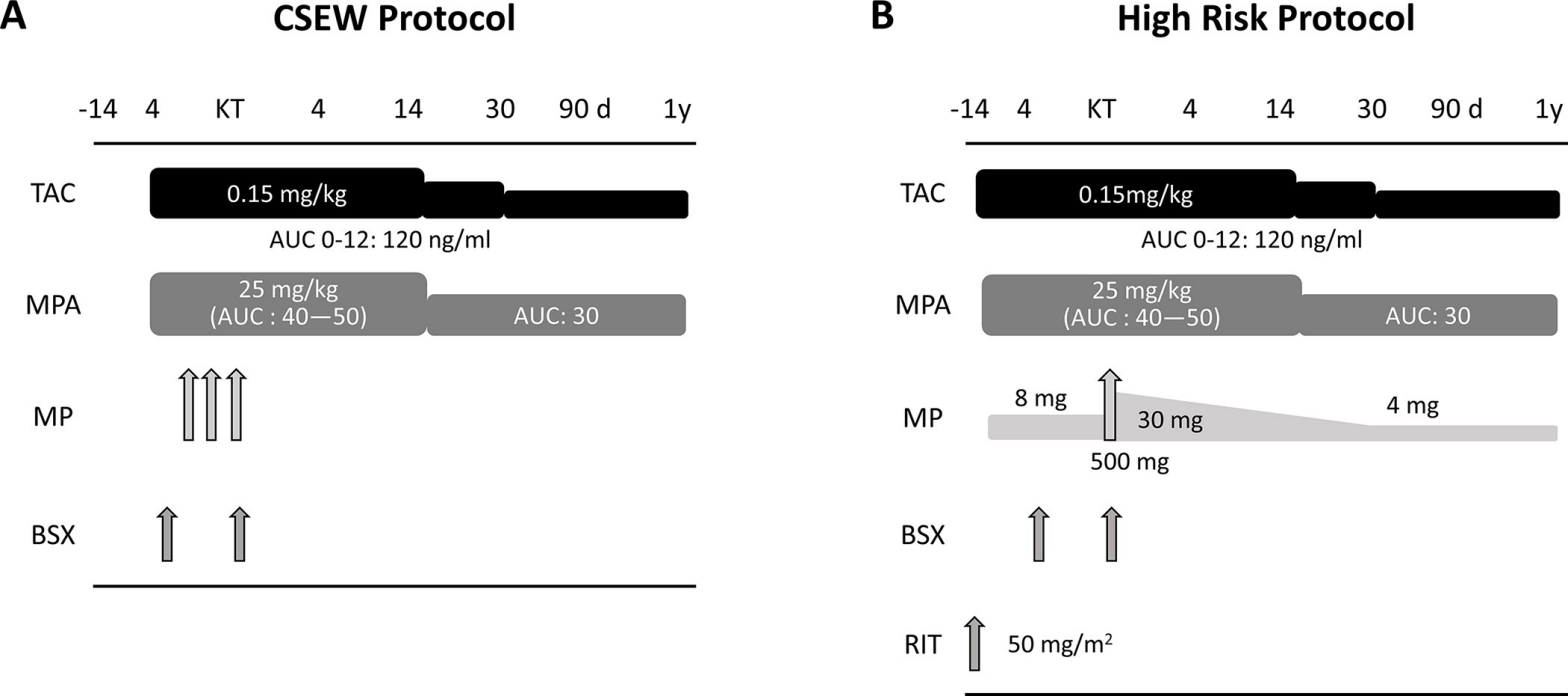
Immunosuppression protocols in our institute. (A) Mycophenolic mofetile (MMF) and tacrolimus (TAC) should be started 4 days before KT. In low risk patients, a corticosteroid, methylprednisolone (MP) is administered as only three injections after KT according to the corticosteroid early withdrawal (CSEW) protocol. (B) In high-risk patients (i.e., ABO incompatible, DSA positive, or FSGS), these two drugs and rituximab (RIT) should be started two weeks before KT.

The immunosuppression regimen is shown in Fig 2. Briefly, all patients were administered TAC twice a day, either formula (TACTD) or TAC extended-release formula (TACER), in combination with mycophenolate mofetil (MMF) and two dosages of the anti-CD25 antibody basiliximab (BSX) (20 mg/body). For low risk patients, the corticosteroid, methylprednisolone, was administered on only three occasions just after KT according to the corticosteroid early withdrawal (CSEW) protocol (Fig 2A). In contrast, chronic administration of methylprednisolone was initiated 14 days before KT and tapered up to 4 mg by 12 mo. in high-risk patients whose grafts were from either ABO blood type incompatible (ABOi) donors, those who were donor specific antibody (DSA) positive, or had the potential to have recurrence of focal segmental glomerular sclerosis (FSGS) (Fig 2B). High-risk patients also received a single dose of the anti-CD20 antibody, rituximab (RIT) (50 mg/m^2^) as reported previously [6]. In 33 recently enrolled patients, everolimus was administered from the beginning of the study as an mTOR inhibitors.

### OGTT and calculation of parameters

A 75 g-OGTT was performed pre-KT and 3, 6, and 12 mo. after KT. According to the American Diabetes Association/World Health Organization guidelines [5], patients whose glucose level at 120 min was < 140 mg/dL, between 140 to 199 mg/dL, or > 200 mg/dL were diagnosed as having normal glucose tolerance (NGT), impaired glucose tolerance (IGT), or DM, respectively. We also measured the immunoreactive insulin (IRI) levels at each sampling point (pre-KT, 30 min, and 120 min after oral glucose intake). These levels were used to calculate ISI and HOMA-β. Because TAC has a stronger effect against beta cell function compared to other diabetogenic immunosuppressants, we also measured blood TAC levels using multipoint blood sampling and analysis of the area under the curve (AUC) at 3, 6, and 12 mo. postoperatively, using 0–12 hour for TACTD, and 0–24 hr for TACER. Our CSEW protocol was an ideal model to minimize the diabetogenic effect of corticosteroids that would affect tissue insulin sensitivity as reported previously [7]. In addition to these parameters, we also investigated the change in the patients’ body mass index (BMI) because weight gain after KT is a crucial factor in the development of PTDM [8].

### Evaluation of diabetogenic factors and estimated GFR (eGFR)

We first investigated the change in the level and severity of glucose tolerance at 12 mo. after KT. Next, we examined the chronological changes in FBS levels and BMI. Third, a 75 g-OGTT was performed, with the increase in blood insulin level 30 min after loading divided by the increase in blood glucose level as an indicator of the initial secretory capacity of additional insulin after a meal. The chronological changes in several parameters such as ISI, HOMA-β, and TAC-AUC were calculated as follows:

**Insulinogenic Index (ISI)** = (IRI_30_–IRI_0_)/(BS_30_–BS_0_). This provides a measure of the acute insulin response to a glucose load that reflects insulin resistance in muscle, with a value of 40% or less considered to indicate the presence of DM.

**HOMA-β** = (IRI_0_ × 360)/ (BS_0_−63). This provides an index of insulin secretion capacity, with a value of 30% or less considered to indicate low insulin secretion.

**Tacrolimus AUC 0–12** = 1.56 × C_1_+ 1.58 × C_3_ + 7.43 × C_6_ + 12.15

**Tacrolimus AUC 0–24** = 1.81 + 4.68 × C_0_ + 3.50 × C_6_ + 10.44 × C_12_

The eGFR at 3 and 12 mo. after KT was calculated as follows:

**eGFR for mal**es = 194 * Scr^-1.094 * age^-0.287

**eGFR for females** = 194 * Scr^-1.094 * age^-0.287*0.739

### Statistical analysis

In this study, we performed statistical analysis, using the R programming language (Version 4.2.2; https://www.R-project.org/). Comparisons between the two groups were analyzed using the Mann-Whitney U-test and Fisher’s exact test. The chronological changes in two and four groups were analyzed using the Wilcoxon signed rank test and Friedman test, respectively. Spearman rank correlation was used to evaluate the association between two variables in the scatter plot. A *P* value < 0.05 was considered to indicate a statistically significant difference in each analysis.

We performed univariate or multivariate logistic regression analysis and built a calibration plot by using the R packages “rms”, “cor”, “Hmisc”, “boot”,. Variance inflation factors of each factor was checked for multicollinearity [9]. The Hosmer-Lemeshow goodness of fit (GOF) test was performed to evaluate the certainty of the prediction model. A significant test statistic with *P*<0.05 indicates the model does not calibrate and the model does not fit between model-predicted and observed values [10]. The bootstrap method was used for making random resampled datasets for internal validation of the calibration plot. Resampled datasets were submitted to the predictive formula 200 times and calculated Brier score. The Brier score indicates model calibration and discrimination, with lower scores showing improved model accuracy [11, 12].

## Results

In this study, every OGTT assessments have been completed on August 31^th^, 2019. Among the 393 patients enrolled in this study, we first compared patients’ characteristics between the eligible patients included in this study (n = 214) and the ineligible patients (n = 179). We found the statistical differences in their Age, HD duration, PKT, and Steroid maintenance (S1 Table).

**Fig 3:**
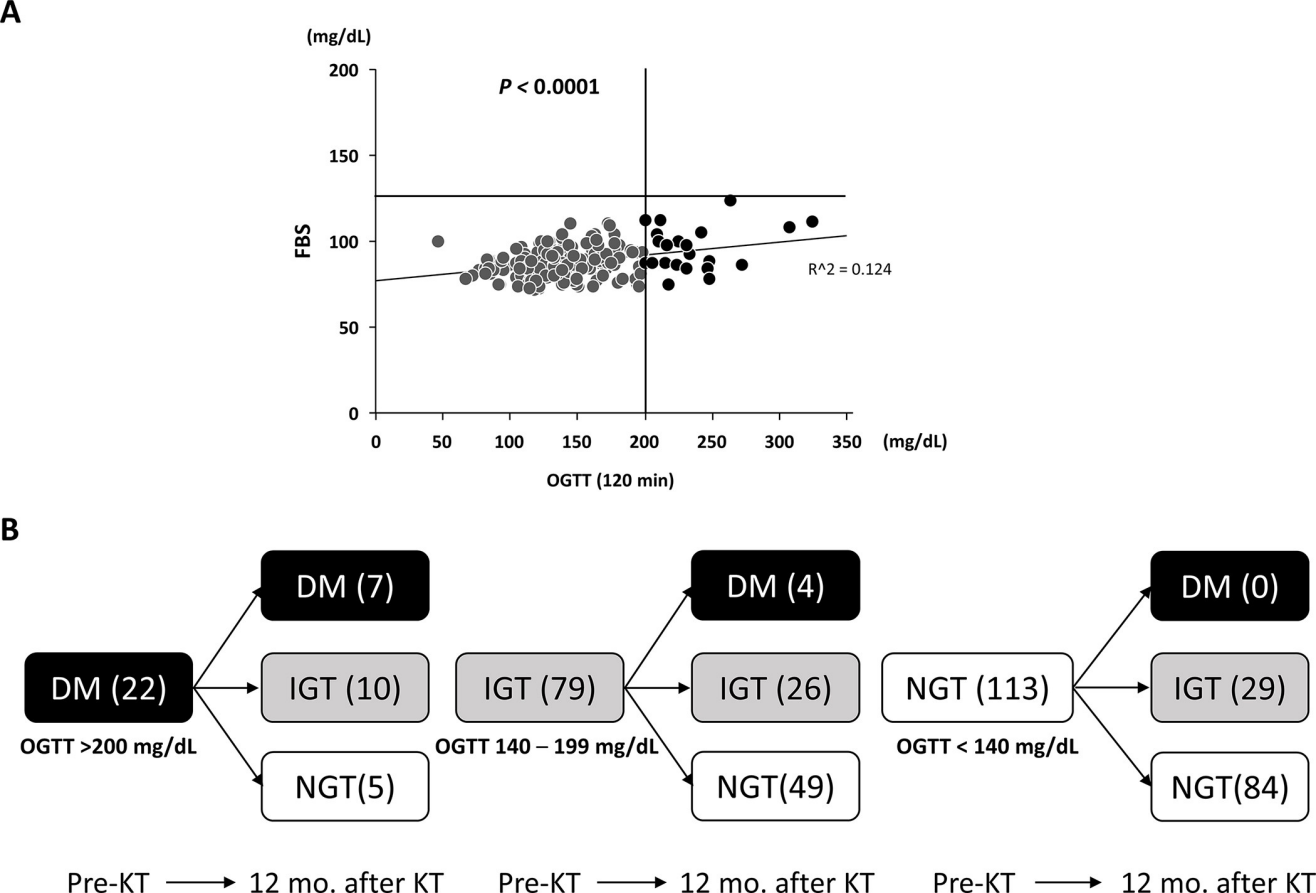
Glucose tolerance status in the patients before and after KT. (A) Preoperative-OGTTs revealed that DM was newly diagnosed in 20 patients who had not been diagnosed with DM by FBS levels only (black circle). (B) Improvement in glucose tolerance at 12 mo. after KT in patients who had been diagnosed with DM or IGT before KT.

Among the eligible patients (n = 214), there were 84 chronic glomerulonephritis (CGN), 64 IgA nephritis, 11 autosomal dominant polycystic kidney disease (ADPKD), 10 focal segmental glomerulosclerosis (FSGS), 6 Henoch-Schönlein purpura nephritis (HSPN), 6 renal sclerosis, 4 congenital anomalies of the kidney and urinary tract (CAKUT), and 29 others as causes of chronic renal failure. There were 158 (73.8%) grafts from ABO blood type comparable (ABOc) donors and 56 (26.2%) from ABOi donors. Graft loss was observed in 14 patients (6.5%): 5 death with functioning graft (DWFG), 4 chronic active antibody-mediated rejection (CAAMR), 2 IgA nephritis recurrence, 1 BK virus infection, and others. Patient with CAAMR were not associated with immunosuppressive regime or OGTT status at 12 mo. after KT. When we reviewed the pre-KT FBS data only, no patient had been diagnosed with DM defined as a FBS > 126 mg/dL. The preoperative-OGTTs showed that DM was newly diagnosed in 22 patients, defined as an OGTT (120 min) > 200 mg/dL (Fig 3A, black circle). Based on the preoperative-OGTT, NGT, IGT, and DM were diagnosed preoperatively in 113 (52.8%), 79 (36.9%), and 22 (10.3%) patients, respectively.

We then classified the patients into two groups (NGT- and IGT+DM group) according to the results of the OGTT. As shown in Table 1, we showed that there was a significant difference between these two groups for Age and ABO-i, but not for the other parameters, i.e. gender, duration of dialysis, BMI, or number of human leukocyte antigen (HLA) mismatches.

**Table 1:** Characteristics of the study population for eligible patients.

	Total (n = 214)	NGT (n = 113)	IGT+DM (n = 101)	*P*-value
Age (median)	45	43	49	0.0033
Male/Female	131/83	71/42	60/41	0.6076
HD duration (mo.)	44	56	30	0.1935
BMI (Kg/m^2^)	21.7	21.9	21.4	0.7380
ABOi	56 (26.2%)	23 (20.3%)	33 (32.7%)	0.0407
HLA mismatch number (median)	3	3	3	0.5269
PKT	85 (39.7%)	44 (39.0%)	41 (40.6%)	0.7606
HbA1c (%)	5.0	5.0	5.1	0.1101
Steroid maintenance	84 (39.3%)	39 (34.5%)	45 (44.6%)	0.1332
EVR (+)	99 (46.3%)	59 (52.2%)	40 (39.6%)	0.0648
TACER (+)	148 (69.2%)	77 (68.1%)	71 (70.3%)	0.7331
Graft loss	14 (6.5%)	8 (7.1%)	6 (5.9%)	0.7366
Mortality	5 (2.3%)	2 (1.8%)	3 (3.0%)	0.5617

NGT: normal glucose tolerance, IGT: impaired glucose tolerance, DM: diabetes mellitus, RHD: hemodialysis, BMI: body mass index, ABOi: ABO blood type incompatible, HLA: human leukocyte antigen, PKT: pre-emptive kidney transplantation, EVR: everolimus, TACER: tacrolimus extended-release formula, The *P*-value was calculated by the logistic regression analysis.

Univariate or multivariate logistic regression analysis were conducted to confirm the relativity between the clinical factors and morbidity of DM at 12 mo. after KT. The analysis excessed as odds ratios and 95% confidence intervals. Multicollinearity was checked using variance inflation factor. We found that Age, FBS, OGTT, ISI, and dyslipidemia were significant or trend to significant independent predictors of PTDM status at 12 mo. after KT (Table 2). There were no significance of primary disease predicting PTDM status at 12 mo. after KT. Multivariate logistic regression analysis showed that preoperative measurements of OGTT and dyslipidemia remained as independent predictors of PTDM at 12 mo. after KT (Table 3). Hosmer-Lemeshow test was performed on the prediction curve. As the result, the chi-square value was 3.241 (*P* = 0.918) and presented good concordance between model-prediction values and actual values. Bootstrap resampling method was used to make internal validation sample (n = 188). Random resampling and refitting were under went 200 times. The Brier score of original data was 0.0296. The mean Brier score of resampled data was 0.0375. The Hosmer-Lemeshow test and internal validation suggested a good agreement between prediction and practicality.

**Table 2:** Univariate logistic regression analysis for predicting DM at 12 months after KT.

Variables	Coef.	SE	Chi-Square	*P*-vale	HR	95% CI
Age	0.069	0.028	6.903	0.0157	1.071	1.017–1.138
Pre-FBS	0.060	0.030	3.725	0.0454	1.062	0.990–1.127
Pre-OGTT	0.029	0.007	21.874	< 0.0001	1.029	1.017–1.045
Pre-ISI	-0.762	0.391	4.392	0.0513	0.467	0.1672–0.959
Pre-dyslipidemia	1.386	0.727	3.956	0.0554	4.000	1.020–19.447

Coef.: coefficient, SE: standard error, HR: hazard ratio, CI: Confidence interval, DM: diabetes mellitus, KT: kidney transplantation, FBS: fasten blood glucose, OGTT: oral glucose tolerance test, ISI: insulinogenic index, The *P*-value was calculated by the logistic regression analysis.

**Table 3:** Multivariate logistic regression analysis for predicting DM at 12 months after KT.

Variables	Coef.	SE	Chi-Square	*P*-vale	HR	95% CI
Age	0.075	0.037	5.309	0.4547	1.777	1.007–1.171
Pre-FBS	-0.014	0.040	2.092	0.7169	0.986	0.910–1.068
Pre-OGTT	0.031	0.010	14.661	0.0012	1.031	1.014–1.054
Pre-ISI	-0.854	0.689	1.012	0.2156	0.426	0.140–1.580
Pre-dyslipidemia	2.224	0.924	6.868	0.0161	9.248	1.718–71.633

Coef.: coefficient, SE: standard error, HR: hazard ratio, CI: Confidence interval, DM: diabetes mellitus, KT: kidney transplantation, FBS: fasten blood glucose, OGTT: oral glucose tolerance test, ISI: insulinogenic index, The *P*-value was calculated by the logistic regression analysis.

PTDM was diagnosed in 11 patients (5.1%) at 12 mo. after KT. Of the 22 patients diagnosed with DM preoperatively, 15 (68.2%) had improved glycemic status to NGT or IGT, while of the 79 patients evaluated as IGT preoperatively, 49 (62.0%) had an improvement to NGT. On the other hand, of the 113 patients evaluated as NGT preoperatively, 84 (74.3%) remained as NGT, with no patient developing DM at 12 mo. after KT (Fig 3B).

**Fig 4:**
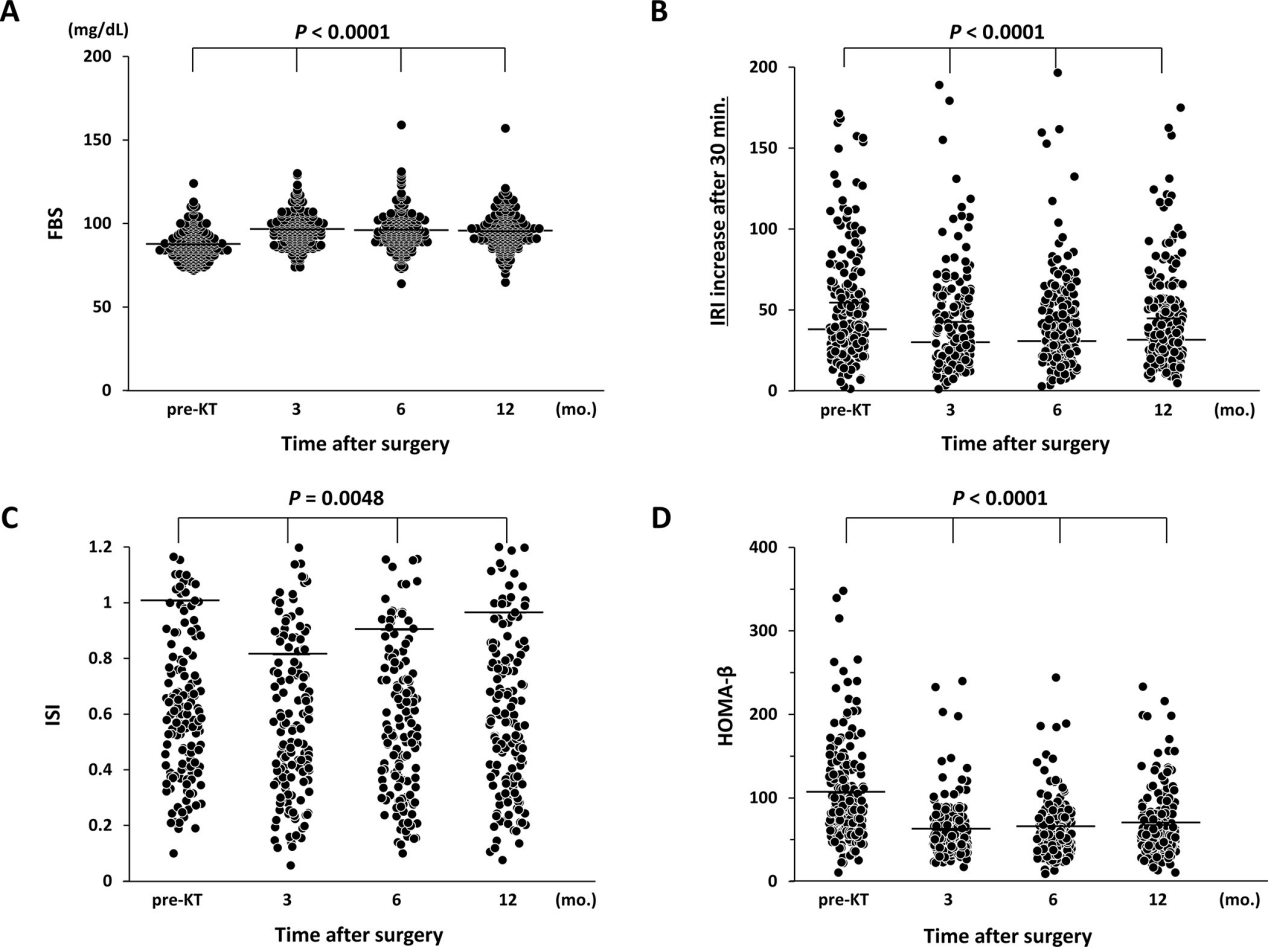
Chronological changes in glucose tolerance parameters: (A) Post-transplant FBS was significantly elevated, although the mean values remained in the normal range. (B,C,D) IRI, ISI, and HOMA-β worsened at 3 mo. but improved over time after KT.

**Fig 5:**
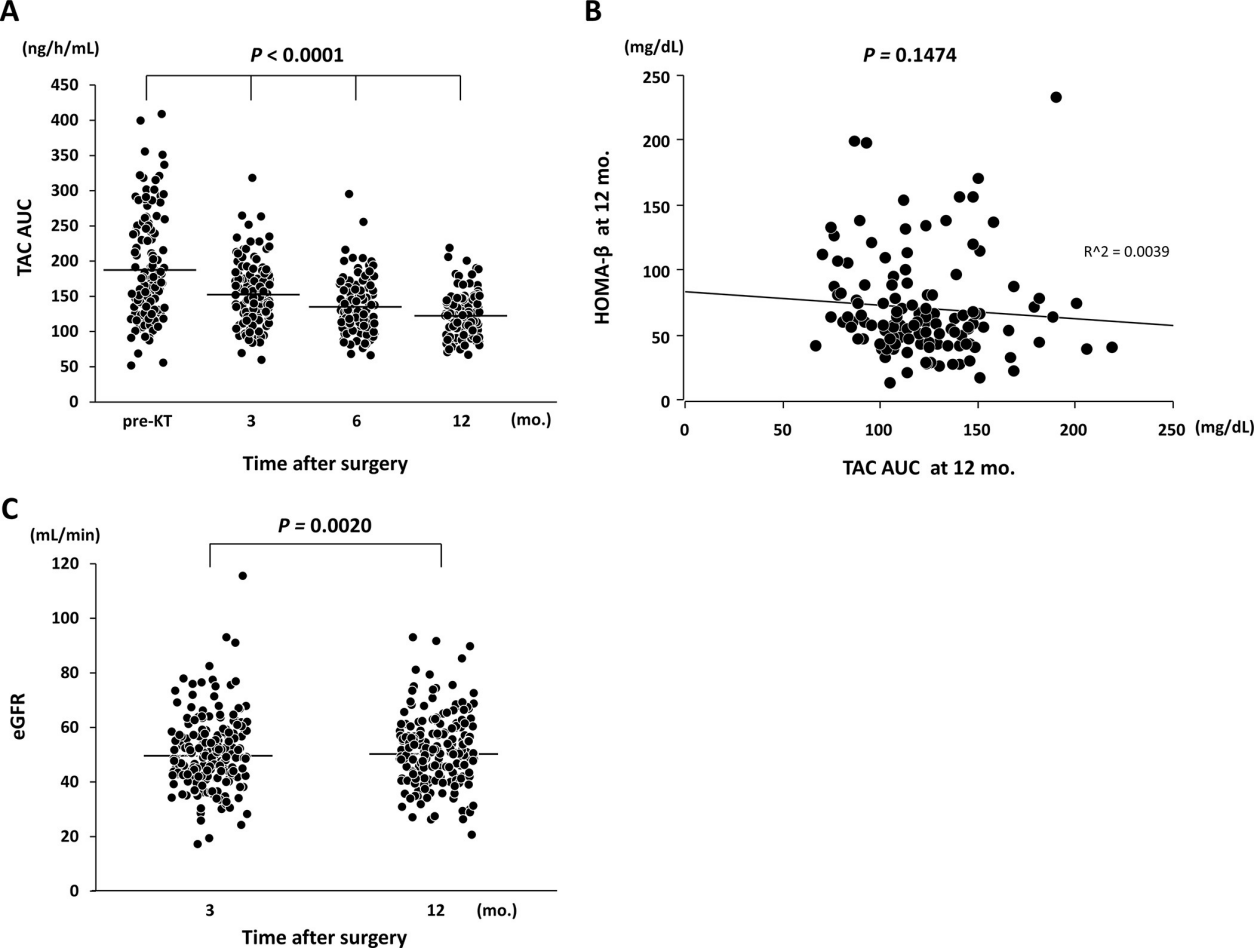
Relationship between TAC-AUC or HOMA-β, and changes in eGFR. (A) TAC-AUC decreased gradually over time after KT. (B) A weak trend of an inverse correlation between HOMA-β and TAC-AUC was observed. (C) There was a significant improvement in eGFR changes between 3 and 12 mo. after KT.

Post-transplant FBS increased gradually with a significant difference (*P* < 0.0001), despite the mean value remaining in the normal range (Fig 4A). The values of IRI, ISI, and HOMA-β were worse at 3 mo. but improved over time after KT with significant statistical values of *P* < 0.0001, *P* = 0.0048, and *P* < 0.0001, respectively (Fig 4B–4D). The median TAC-AUC at 12 mo. after KT was 119.4 ng/mL (range, 67–219 ng/mL), while the median trough value measured at that time was 3.3 ng/mL (range, 1.6–8.3 ng/mL). The study also showed no significant difference in TAC-AUC concentration between the glucose intolerance groups (NGT vs. IGT+DM) at 12 mo. after KT (123.6 vs. 113.5 ng/mL, *P* = 0.3357). TAC-AUC was highest at pre-KT and decreased gradually over time following the KT (*P* < 0.0001) (Fig 5A). In addition, a weak trend towards an inverse correlation was observed between HOMA-β and TAC-AUC (*P* = 0.1474, R^2 = 0.0039) (Fig 5B), whereas there was no correlation between ISI and TAC-AUC (*P* = 0.8939). The study showed that a significant improvement in median eGFR occurred between 3 and 12 mo. after KT (48.8 vs. 50.2 mL/min, *P* = 0.0020).

**Fig 6:**
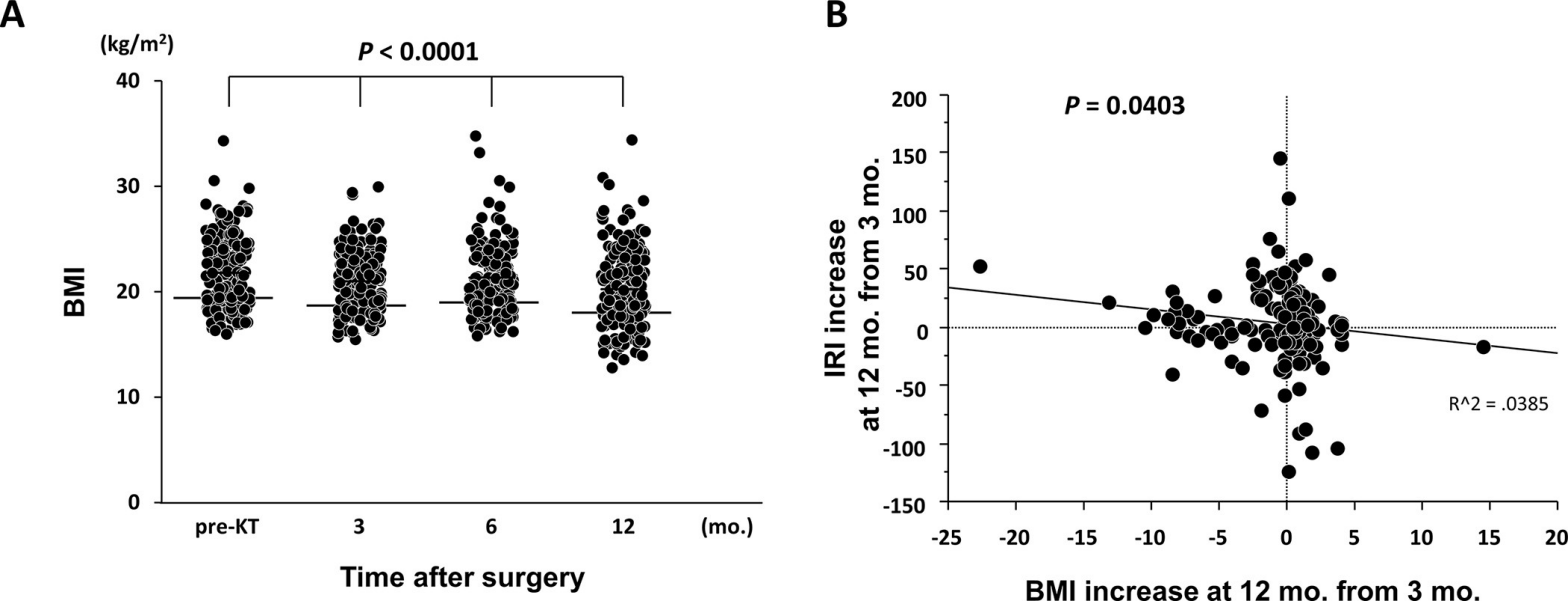
The relationships between BMI and glucose tolerance parameters. (A) A significant decrease in BMI occurred over time after KT. (B) The inverse correlations between IRI- and BMI increases between 3 and 12 mo. after KT.

The transplant recipients showed a significant decrease in BMI over time, with a median value of 19.9 kg/m^2^ at 12 mo. after KT (*P* < 0.0001) (Fig 6A). As shown in Fig 6B we observed a significant inverse correlation between IRI and BMI increase between 3 to 12 mo. after KT (*P* = 0.0403, R^2 = 0.0385). There was no correlation between HOMA-β and BMI increase from 3 to 12 mo. after KT (*P* = 0.3503).

## Discussion

The incidence of PTDM has been reported to be 10–40% [2, 4], whereas in Japan, the incidence of PTDM at 12 mo. after KT was reported to be relatively low at 13% [13]. However, that study had several limitations. For example, although all the patients in the study underwent a 75g-OGTT preoperatively, a routine OGTT was not carried out at the follow-up clinic after KT, and therefore, the definition of PTDM was not appropriate. In the present study, the prevalence of PTDM at 12 mo. in the transplant recipients was extremely low at 5.1%. We used an OGTT to evaluate glucose tolerance because it is recognized as the gold standard for evaluating impaired glucose tolerance [14].

However, in the current study, glucose intolerance was simply assessed by an OGTT in order to determine the precise incidence of PTDM. Therefore, the possibility of underestimating glucose intolerance in our cohort was considered to be very low. In addition, our study showed that 65% of patients diagnosed with DM in the preoperative-OGTT were classified as having IGT or NGT at 12 mo. after KT. This indicated that their glucose intolerance had improved after KT. Likewise, 63% of patients who had been diagnosed with IGT preoperatively improved to NGT at 12 mo. after KT. Taken together, these results are better than those reported in previous studies [15].

Age, family history, hepatitis C virus, BMI > 25 kg/m^2^, a history of low FBS prior to KT, and genetic factors have been reported to be pre-KT risk factors for PTDM [4, 16, 17]. Based on the results of the multivariate logistic regression analysis of our cohort, preoperative measurements of OGTT and dyslipidemia remained as independent predictors of PTDM at 12 mo. after KT. This suggested that precise evaluation of pre-KT glucose tolerance might be an important factor for predicting PTDM [9, 18, 19].

Post-KT risk factors for PTDM have been reported to include immunosuppressive agents such as mTOR inhibitors and CNI, cytomegalovirus infection, graft rejection, and the following phenomena [20]. After KT, there is basically a decrease in blood insulin concentration due to improved renal function and impaired insulin secretion caused by tacrolimus [21, 22]. These phenomena may result in worsening of both FBS and OGTT post-load plasma glucose level [23]. The fact that HOMA-β improved from 3 mo. after KT with a weak inverse correlation observed between HOMA-β and TAC-AUC at 12 mo. implies that glucose tolerance after KT might be improved due to long-term maintenance of a low level of TAC-AUC, despite our finding that a low TAC-AUC had a limited effect on the level of glucose tolerance measured 3 mo. after KT. The median AUC at 12 mo. after KT was 119.4 ng/mL, which indicated good control at the target level of 120 ng/mL of our institution. However, the median trough value measured at that time was 3.3 ng/mL, which was lower than the normally recommended trough value of around 5–8 ng/mL [24]. Sharif et al. [17]. reported that tacrolimus exacerbated insulin resistance and had a positive effect of minimizing CNI on diabetes after KT [3, 16]. Kim et al. also reported that tacrolimus destroyed pancreatic beta cells, worsened insulin resistance, and that it was more likely to result in PTDM. They also reported that tacrolimus use was more likely to cause glucose intolerance than cyclosporine, indicating that the addition of everolimus may reduce the dose of tacrolimus and the occurrence of PTDM in addition to avoiding acute rejection [7, 18, 25]. Our study did not show an improvement in ISI, which is a measure of the capacity of insulin secretion. Because ISI is thought to deteriorate following the administration of tacrolimus [26], strict and minimal control of tacrolimus blood levels might have contributed to an improvement in ISI in the patients at our institution. Furthermore, we found a significant improvement in eGFR after KT, suggesting that no adverse effect had occurred in renal function as a result of minimal control of tacrolimus blood levels.

The majority of previous studies evaluated and adjusted tacrolimus doses using trough values but not strictly by AUC as used in this study. In the future, it might be standard practice to adjust tacrolimus dosage by AUC.

Aksoy et al. reported that a weight gain of 6 to 10 kg was common 6 mo. after KT [27]. A large study of KT patients also showed that the risk of PTDM increased in overweight patients (BMI > 25 kg/m^2^) and was evident in obese subjects (BMI > 30 kg/m^2^) [28]. Another study showed that the risk of PTDM increased linearly with each kilogram over 45 kg [19]. Bzoma et al. also reported that a high BMI was a risk factor for PTDM [29]. In contrast, as shown in the results, the patients in our study showed a significant decrease in BMI from pre-KT to 12 mo. after KT. We also found weak inverse correlations between IRI and an increase in BMI between 3 and 12 mo. after KT, suggesting that an increase in BMI from 3 mo. after KT might cause worse glucose intolerance. These results may the reason why we achieved a low incidence of PTDM in our institution. Our hospital individualizes lifestyle interventions for each patient, which may have contributed to this improvement in glucose tolerance.

### Limitations

This study had several limitations as it was a prospective observation design carried out at a single center that included a small number of cases. It seems related to institutional factors regarding limited immunosuppression regimen and a well-managed process for identifying and treating patients with IGT/DM. Therefore, the generalizability of the study limited. In our cohort, there were 179 ineligible patients due to incomplete protocol testing (n = 124) or having cadaveric KT (n = 55). The age, HD duration, PKT ratio were significantly higher, longer, and smaller in the ineligible patients compared to eligible patients, because cadaveric KT was included in the 30.7% of ineligible patients (S1 Table). Steroid maintenance ratio was also smaller in the eligible patients compared to the ineligible patients due to unknown reason. These differences might affect the analyses of the eligible patients.

## Conclusion

In our cohort, the real incidence of PTDM at 12 mo. after KT was extremely small in comparison with that reported by previous similar studies. Moreover, glucose tolerance could be improved after successful KT in patients preoperatively diagnosed with DM or IGT.

Our early corticosteroid withdrawal or steroid minimization protocol using tacrolimus, by keeping a low level of diabetogenic tacrolimus and decreasing BMI after KT by individualized lifestyle interventions contributed to an improvement in post-KT glucose tolerance.

## Supporting information

S1 Table Characteristics of the study population for all patients. The characteristics for all enrolled patients including 214 eligible and 179 ineligible patients.
(DOCX)

S1 Dataset (XLSX)
