# Supplementary material for: Low dose tacrolimus exposure and early steroid withdrawal with strict body weight control can improve post kidney transplant glucose tolerance in Japanese patients
Source: PLoS One. 2023 Oct 11;18(10):e0287059. doi: 10.1371/journal.pone.0287059 (PMC10566682; doi:10.1371/journal.pone.0287059)
Supplement: S1 Table — The characteristics for all enrolled patients including 214 eligible and 179 ineligible patients. (DOCX) [file pone.0287059.s001.docx]

Table S1 Characteristics of the study population for all patients

|  | Total  (n = 393) | Eligible  (n = 214) | Ineligible  (n = 179) | *P*-value |
| --- | --- | --- | --- | --- |
| Age (median) | 48 | 45 | 52 | 0.0001 |
| Male/Female | 238/155 | 131/83 | 107/72 | 0.7714 |
| HD duration (mo.) | 71 | 44 | 105 | <0.0001 |
| BMI (Kg/m^2^) | 21.8 | 21.7 | 22.1 | 0.9210 |
| ABOi | 99 (25.2%) | 56 (26.2%) | 43 (24.0%) | 0.6482 |
| HLA mismatch number (median) | 3 | 3 | 3 | 0.4722 |
| PKT | 122 (31.0%) | 85 (39.7%) | 37 (20.7%) | <0.0001 |
| HbA1c (%) | 5.1 | 5.0 | 5.2 | 0.0488 |
| Steroid maintenance | 186 (39.7%) | 84 (39.3%) | 102 (57.0%) | 0.0005 |
| EVR (+) | 181 (46.1%) | 99 (46.3%) | 82 (45.8%) | 0.9287 |
| TACER (+) | 254 (64.6%) | 148 (69.2%) | 106 (59.2%) | 0.8316 |
| Graft loss | 30 (8.3%) | 14 (6.5%) | 16 (8.9%) | 0.3729 |
| Mortality | 15 (3.8%) | 5 (2.3%) | 10 (5.6%) | 0.0940 |
